# The Metric System of Weights and Measures

**Published:** 1886-01-20

**Authors:** Oscar Oldberg


### THE METRIC SYSTEM OF WEIGHTS AND MEASURES.

BY OSCAR OLDBERG, PHARM. D.

The metric system is based upon the METER, which is the standard unit of *length* of that system, and equal to 39.370432 inches, or about 10 per cent. longer than the yard.

The metric unit of *fluid measure* is the LITER—the cube of 1-10 meter, or 1000 cubic-centimeters—equal to about 34 fluid ounces.

The metric unit of *weight* is the GRAM, which represents the weight of one cubic-centimeter of water at its maximum density. It is equal to about 15 grains.

One CUBIC CENTIMETER is equal to about 16 minims.

In writing prescriptions it is sufficiently accurate and safe to consider 1 gram as exactly equal to 15 Troy grains, and to consider 1 cubic-centimeter as equal to 15 minims.

We accordingly have—

1 gram equal to 15 Troy grains.
1 Troy grain equal to 1-15 gram.
1 cubic-centimeter equal to ¼ fluid drachm.
1 fluid drachm equal to 4 cubic-centimeter.

Hence—

1. To convert Troy grains into grams, or minims into cubic-cetimeters :

*a.* Divide by 10, and from the quotient subtract one-third, or, *b*, Divide by 15 ; and

2. To convert Apothecaries' drachms into grams, or fluid drachms into cubic-centimeters, multiply by 4.

In writing prescriptions the "Gram" (abbreviated "Gr.") and "Cubic-centimer" (abbreviated "C. c.," which may be called "fluigram," and written "f. Gm"), only, should be used.

All other terms, and units, and prefixes, used in the metric system, may be wholly ignored by the physician and the pharmacist.

Example of a Metric Prescription :

| | | | | | |
|---|---|---|---|---|---|
| ℞. Hydrarg. Chloridi. Corros. .......... | 0 | 25 Gm. | : 6½ | = 4 gr. |
| Potassii Iodidi ...................... | 10 | 00 Gm. | : 4 | = 2½ ʒ |
| Aquæ ............................ | 100 | 00 C. c. | : 4 | = 25 ʒ |
| Tinct. Cinch. Comp. ................ | 100 | 00 C. c. | : 4 | = 25 ʒ |

Mix.

The use of the decimal line prevents possible errors.

